# A Qualitative Study of the Theory Behind the Chairs: Balancing Lean-Accelerated Patient Flow With the Need for Privacy and Confidentiality in an Emergency Medicine Setting

**DOI:** 10.2196/11714

**Published:** 2019-02-06

**Authors:** Elaine Zibrowski, Lisa Shepherd, Richard Booth, Kamran Sedig, Candace Gibson

**Affiliations:** 1 Health Information Science Faculty of Information & Media Studies University of Western Ontario London, ON Canada; 2 Department of Medicine Division of Emergency Medicine University of Western Ontario London, ON Canada; 3 Arthur Labatt Family School of Nursing University of Western Ontario London, ON Canada; 4 Department of Computer Science University of Western Ontario London, ON Canada; 5 Department of Pathology University of Western Ontario London, ON Canada

**Keywords:** Lean health care, emergency medicine, privacy and confidentiality, work intensification, qualitative research

## Abstract

**Background:**

Many emergency departments (EDs) have used the Lean methodology to guide the restructuring of their practice environments and patient care processes. Despite research cautioning that the layout and design of treatment areas can increase patients’ vulnerability to privacy breaches, evaluations of Lean interventions have ignored the potential impact of these on patients’ informational and physical privacy. If professional regulatory organizations are going to require that nurses and physicians interact with their patients privately and confidentially, we need to examine the degrees to which their practice environment supports them to do so.

**Objective:**

This study explored how a Lean intervention impacted the ability of emergency medicine physicians and nurses to optimize conditions of privacy and confidentiality for patients under their care.

**Methods:**

From July to December 2017, semistructured interviews were iteratively conducted with health care professionals practicing emergency medicine at a single teaching hospital in Ontario, Canada. The hospital has 1000 beds, and approximately 128,000 patients visit its 2 EDs annually. In response to poor wait times, in 2013, the hospital’s 2 EDs underwent a Lean redesign. As the interviews proceeded, information from their transcripts was first coded into topics and then organized into themes. Data collection continued to theoretical sufficiency.

**Results:**

Overall, 15 nurses and 5 physicians were interviewed. A major component of the Lean intervention was the construction of a three-zone front cell at both sites. Each zone was outfitted with a set of chairs in an open concept configuration. Although, in theory, professionals perceived value in having the chairs, in practice, these served multiple, and often, competing uses by patients, family members, and visitors. In an attempt to work around limitations they encountered and keep patients flowing, professionals often needed to move a patient out from a front chair and actively search for another location that better protected individuals’ informational and physical privacy.

**Conclusions:**

To our knowledge, this is the first qualitative study of the impact of a Lean intervention on patient privacy and confidentiality. The physical configuration of the front cell often intensified the clinical work of professionals because they needed to actively search for spaces better affording privacy and confidentiality for patient encounters. These searches likely increased clinical time and added to these patients’ length of stay. We advocate that the physical structure and configuration of the front cell should be re-examined under the lens of Lean’s principle of value-added activities. Future exploration of the perspectives of patients, family members, and visitors regarding the relative importance of privacy and confidentiality during emergency care is warranted.

## Introduction

### Background

The provider-patient relationship is the foundation of medicine, and this relationship revolves around trust. As part of a trusting relationship, a patient must have faith that any information exchanged during their encounter with a physician or nurse will remain private and confidential [1,2]. Although privacy and confidentiality share some ideas, these 2 concepts have distinct definitions. Privacy has physical, decisional, and informational dimensions. Regarding these dimensions and medical care, a patient should not experience any unnecessary or embarrassing exposures of their body. A patient should also be free to make informed decisions regarding their care without facing undue pressure or interference from another individual, and they should entrust that any information collected during their medical care will be kept confidential [3-5]. Violations of confidentiality can be intentional or unintentional [3,6]. Intentional violations occur when a professional directly communicates a patients’ information to an unauthorized person. Unintended violations arise when conditions are inadvertently created that enable an unauthorized individual to see or hear information about a patient. Intentional violations or failure to adequately protect a patients’ personal health information may result in an investigation or audit from a professional regulatory organization, with potential consequences including disciplinary action [1,2].

An emergency department (ED) is considered to be one of the most complex environments in which to deliver patient care [5,7-10], and although reviews by Ulrich et al have highlighted a dearth of research in this area, there are some indications that the design and layout of an ED can increase the vulnerability of patients to breaches of their physical and informational privacy and confidentiality [11,12].

Mlnek and Pierce asked trained observers to record patients’ names plus their diagnosis/reason for treatment while they were sitting in a triage chair or empty treatment areas of a hospital-based ED in the United States. From the triage chairs, observers recorded the names of 81% (26/32) of patients and the diagnosis/reason for treatment for 56% (18/32). Both elements were recorded for 53% (17/32) of triaged patients. Observers noted that when they were stationed in treatment areas with curtains, they were able to hear “almost everything” that occurred in adjacent areas. When curtains were left open in other rooms, observers were also able to craft detailed notes about medical procedures they saw being performed on patients. The authors noted that no privacy breaches were recorded when observers were stationed in empty patient rooms with solid walls [13].

In another American study, Zhang et al asked an observer to record the ambient conversation while they were seated near a nurses’ station and in some empty patient rooms of hospital-based ED. Thematic analyses of transcripts prepared from the recordings revealed that nursing station conversations predominantly revolved around patient care (86% of content; 95% CI 68.7-94.7). Although patient names were not heard on the nurses’ station recordings, other details including individuals’ medical and social histories, physical examination results, and diagnoses were audible. The authors noted that although 44.8% (95% CI 17.7-62.2) of the conversations that were recorded from patient rooms revolved around clinical topics, these contained very little patient-related information [14].

Karro et al reported that 45.1% (106/235) of patients treated by an Australian ED had been involved in a privacy incident at an Australian hospital-based ED. Overall, 41% of patients (95% CI 35-47) revealed they had overheard information involving another patient, and 15% (95% CI 11-21) sensed other members of the public had overheard conversations related to their care. Overall, 10% (95% CI 06-14) admitted they saw another patient’s body, and 4% (95% CI 02-07) felt their body was exposed. Patients treated within walled cubicles were significantly less likely to overhear information about another patient (*P*<.002) and felt their information would be less likely to have been heard by an unauthorized person (*P*=.06) [15].

Finally, patient surveys from Barlas et al in the United States and Lin and Lin from Taiwan explored whether patients’ perceptions of privacy and confidentiality impacted how they interacted with members of their ED care team. Patients in both of these study cohorts admitted that due to a perceived lack of privacy and confidentiality, they withheld aspects of their medical history or had refused parts of their physical exam (Barlas, a total of 3.7% [4/108] of patients; Lin, 21.2% [23/108] of patients withheld aspects of history; and 19.4% [21/108] of patients refused parts of exam). These studies appeared to put forth different viewpoints regarding the degree of effort made by members of the care team to circumvent breaches. Although Barlas reported that 85.2% (92/108) of patients in their study perceived that ED staff showed respect for their privacy, Lin and Lin concluded, “in our opinion, the most important factor influencing patient privacy was lack of vigilance in the ED” [16,17].

Many EDs had used Toyota’s Lean methodology to guide the restructuring of their practice environments and patient care processes [18,19]. As part of its focus on continuous improvement, Lean asks an organization to rethink how they are delivering what is of value to their customers [18,20-25]. Organizational processes are broken down and examined regarding whether they contribute value-adding activities [18,19]. Value-added activities are those that work toward satisfying customer needs. Conversely, nonvalue activities detract an organization from achieving its goals and waste time and resources including personnel and physical space. By removing nonvalue activities, the Lean method asserts that an organization will be able to streamline its processes and deliver what customers want at a faster pace [5,20-22,25].

### Objective

Given that Lean health care focuses on the enhancement of patients’ experiences, it would seem to follow that when a Lean intervention is evaluated, it should include some examination of how it potentially affected patient privacy and confidentiality. However, reviews by Holden regarding the implementation of Lean interventions in EDs and reviews by Moraros et al of the effects of Lean interventions across multiple medical settings, have suggested that the topic of privacy and confidentiality has not been a priority in the Lean health care discourse [18,24]. None of the articles that were part of these 2 reviews looked at the potential impact of Lean-driven changes on patient privacy and confidentiality. Moraros’ review also presented primary analyses of patient satisfaction data that were gathered by hospitals in the Canadian province of Saskatchewan. Saskatchewan is recognized to have undergone the largest Lean health care transformation in the world, and this included restructuring of provincial EDs [19]. Although the Saskatchewan analyses included multiple indicators of provider-patient communication, measures specifically tied to patients’ privacy and confidentiality were not presented. Moreover, we were not able to locate a study about Lean and patient privacy and confidentiality through our searches of the published literature.

If professional regulatory organizations are going to require that physicians and nurses interact with their patients privately and confidentially, we need to examine the degrees to which their practice environment supports them to do so. The purpose of this study was to explore how a Lean intervention affected the ability of emergency medical professionals to optimize conditions of privacy and confidentiality for patients under their care.

## Methods

### Study Design

The findings reported by this study arose from data collected as part of a realist grounded theory study that examined the impact of a Lean intervention undertaken by 2 EDs from a single teaching hospital in Ontario, Canada [26]. The hospital has 1000 beds, and about 128,000 patients seek treatment from the 2 adult EDs annually. In 2013, in response to poor ED wait times, the hospital introduced extensive changes to the physical practice environments and patient care processes at both adult sites. The changes were anticipated to improve the efficiency of the ED, and in turn, this would reduce the wait times experienced by patients.

From July to December 2017, emergency nurses and physicians who practiced at either ED were sent emails inviting them to consider participating in a single, semistructured interview. These emails were sent on behalf of the study by the administrative office that manages the ED sites, and professionals were asked to reply to EMZ’s confidential university email account. The administrative office was not made aware of the participants’ identities. Interviews were audio-recorded for transcription into verbatim electronic documents by a professional transcription service. Participants received a Can $20 gift card as an honorarium. University and hospital-level research ethics boards approved the study’s protocol.

The study followed a constant comparative approach that is consistent with grounded theory methodology [27-29]. Using a semistructured format, the interview probed the ED environment, both before and after the Lean intervention, including the physical configuration of space, organization of patient flow, clinical workflow for physicians and nurses, opportunities for professionals to collaborate during patient care, the motivation for restructuring the ED, and the processes that were involved in the Lean intervention.

### Data Analysis

After each interview, field notes were prepared about the dialogue that occurred with the professional, and these notes were reviewed alongside the interview’s prepared transcript. With the use of MAXQDA software (Version 11.2.5, VERBI Software, Sozialforschung GmbH, Berlin, Germany), information from the transcripts was first read line-by-line and was coded into a set of categorical topics. Categorical coding continued alongside data collection, and information from new interviews was successively compared with the existing set of codes. Through the repeated review of the interview transcripts and evolving coding, the categorical topics were organized into themes. Data collection continued to theoretical saturation of meaning at which point we felt that the amount of information that was gathered from the interviews was sufficient to support our exploration of participants’ perspectives and that any additional interviews were not likely to introduce major modifications to our understanding of the data gathered in our study [30,31]. For our research, we sensed theoretical sufficiency after 20 interviews.

## Results

### Demographics

Overall, 15 nurses and 5 emergency physicians were interviewed, and 18 of these individuals had been practicing emergency medicine for at least 10 years. Interviews lasted, on average, 53.8 min (SD 11 min), and the corpus of transcripts contained a total of 171,592 words of content.

### Themes

All of the health care professionals who were interviewed during this study spoke in detail about their experiences providing medical care to patients and interacting with their family members and visitors within a particular area of the restructured ED, the front cell. The construction of the front cell was a major element of the Lean redesign. The front cell tends to be a very busy area of the restructured ED because it receives all of the patients that are flowed forward from triage. The experiences of nurses and physicians in the front cell revolved around 3 themes: the theory behind the chairs, too many people in the front cell, and how we work (around) to try to preserve our patients’ privacy. After a description of the physical configuration and patient care processes used in the front cell, the 3 themes will be unpacked with anonymized quotes to ground, and enrich, our findings with the voices of our study participants.

### The Front Cell

The front cell of the ED was separated into 3 zones, and triaged patients were directed to one of these. Each zone had 3 stretcher beds and 6 chairs, and the primary assessment nurse, who manages patient flow, made the initial decision of whether a patient was sent to a stretcher bed or front chair. Although the stretcher beds had surrounding curtains, the chairs did not. Instead, the chairs were located together in an open concept configuration and were spaced in a side-by-side array. Before its restructuring, the ED did have some chairs available for patients, but these were situated away from treatment areas and spaces where nurses and physicians completed their charting.

### The Theory Behind the Chairs

Participants explained that the theory behind equipping the front zone with sets of patient chairs arose from an accepted idea that ambulatory patients should remain ambulatory:

The point of chairs is to be able to keep upright patients upright. So, if you can walk and you do not need a stretcher, per se, because your medical condition does not need you to be on a stretcher, they would seat you in a chair. A patient who was young, healthy but just needs a quick exam, belly exam, something like that, or someone with an isolated orthopedic injury. They would be able to take the patient from the chairs into that first bed, see them there and then put them back into the chairs for a plan.

N101

If you were a gallbladder, you need a bed. If we triage you and the assessment nurse has done your blood work, and it comes back, and it is fine, you'll sit in the chair until you get to an examination table for the doctor to be able to do a full exam. If you were sweating profusely, pale, not doing well, we would have you still in one of those stretchers until we get you pain-free. We may be able to move you over to the Rapid Assessment Zone, or to the middle bubble, while we get ultrasounds and that. If you are doing really well and you look well enough to sit in a chair and weren't in crisis, then you would sit in a chair, continue to give you medication, and go from there.

N112

### Too Many People in the Front Cell

Several participants clarified that the ED seemed to have drifted away from its original plan to designate the front cell as a patient-only area. In the original plan, under certain circumstances would a family member or friend be allowed to accompany a patient forward to the front cell after triage. For example, if a patient had a cognitive issue or they required an interpreter, 1 person would be allowed to remain with the patient. Although interviewees empathized with individuals’ desire to be with a loved one or friend while that person was being cared for in the ED, they noted that over time, the front cell had become, in essence, a secondary waiting room. As this nurse noted, it was common for patients to bring one or more people with them into a front zone:

People get rather annoyed if family members can’t stay with their loved ones, which I understand. I always try and say, “We don’t need to have five family members for the one person.”

N107

During times when family and visitors accumulated in the front zones, interviewees noted fewer chairs were available for patients, family members crowded around stretcher beds, hallways became congested, ambient noise levels increased, and as this nurse and emergency physician explained, it was often difficult for a health care professional to work in a front zone while their family members and visitors were also present:

I understand that they are worried, and they are concerned about their family members, but I have actually had put it to them, and I said, “If anything ever happened to your family member, I can't get to them. I'm not going to be tripping over chairs or you to do my job. Please trade off any time you like, but I can only have one [of you here].”

N110

It’s become like a waiting room. And even at the [stretcher] bedside, it's a small geographical space. And there are many times I'll open the curtain to try to walk in, and there will be three or four visitors with the patient. In a small area where you’re trying to provide such rapid care, you cannot do it with visitors there. It was initially intended that you would do your care in the front bubble [without visitors]. Once the patient was moved, visitors or family would be allowed to come into the areas where they have been moved. The general public hasn't accepted that. And that space has just never been designed to allow for that.

P204

Moreover, some participants noted that the public often ignored the hospital’s request that they refrain from using mobile devices in the ED, and you could see people using their mobile phones while they waited in a front zone.

Interviewees explained that during periods of high patient volume and slowed access to the stretcher beds in the front zones plus the accumulation of patients, family members, and visitors in the front cell synergized to increase the likelihood that a patient would interact with a health care professional in the presence of other members of the public. Professionals were not comfortable with this situation as described by these 2 nurses:

I like the idea of either putting them in the chairs to wait to come into a spot [stretcher bed]. Say, all our spots are full, there are a few more people that are appropriate, trade them out. That is fine. Or they have had their lab work done, and they are just waiting for results, they are stable, put them into the chair and wait for the results. I am okay with that. It’s the people that are getting seen by physicians or nurses in the chairs. I don’t like that. I don’t like assessing a patient in a room full of a bunch of other people, asking them personal information questions, things like that.

N110

You could have patients in chairs surrounded by strangers beside you inches away, and a doctor is asking you questions. Yeah, or even if it’s just assessing your foot, people are watching that, they’re right there. And even in the stretchers, you can hear everything that’s going on behind those curtains. The historical set up though we’ve had curtains, so there’s always been some lack of confidentiality, but with the cluster of chairs where they are out in the open now. Oh, it’s terrible. I think about if I was a patient how I would feel with that and I would probably put a complaint in because there is no privacy there.

N109

Concerns were raised regarding the impact of the departments’ push-forward model on the front chair environment. Physicians explained that as the model prioritized the continual flow of patients from triage, this meant that at any given moment, it was feasible for individuals with varied medical needs to be seated together. They cautioned that although a patient may be alert and mobile when they are assessed at triage, and therefore, would be eligible to be pushed forward into a chair, it should not be assumed that the individual was experiencing a minor medical complaint. Although some physicians recalled instances where they felt a triaged patient should have been directed to a stretcher bed rather than a chair, they also acknowledged there was not always an available alternative:

I honestly feel we have to put people in chairs that should not be in chairs. But the alternative is, they wait in the waiting room [by registration]. So, I’ll say to patients, “I’m sorry that you have to be in that spot [a front chair], but it’s either that or you don’t get seen at all.” And people understand that equation, but it doesn’t mean they’re happy about it, particularly if they’re not feeling well.

P202

Further, as these 2 physicians highlighted, there were medical contexts where doctors anticipated it could be especially uncomfortable for a patient to have to interact with them while they were sitting among other people:

The chairs are where I have difficulty because there will be multiple patient types in chairs. You might have two psychiatry patients, you could also have someone waiting on blood work, and you could have someone that has a sore foot in the chairs. My perception is that most patients don’t like to be talked to in front of a bunch of other people. Of course, it depends on why you are there in the first place. If you have a cut on your thumb, you may very well not mind talking about it in front of other people.

P200

Sometimes you’re asking some pretty uncomfortable questions to people. Like, you [the emergency physician] need to know this, or you don’t know this. So for them [patient] to, sort of, to be quizzed, or asked, or somewhat berated sometimes in front of a room, and then to have to go see that next person 10 feet away, that person knows exactly what’s going on. Whereas in the old system, you had that privacy, and to discuss issues about patients that, you know, that person had chlamydia, gonorrhea, or something else. That’s probably not the nicest conversation in a room full of 50 people.

P201

### How We Work (Around) to Try to Preserve Patient Privacy

We have previously described that managing high patient volumes in the ED commonly involved moving patients around the 3 cells of the reconfigured ED [26]. When interviewees elaborated on reasons underlying these moves, they explained that they would often ask a patient to move out of a front chair and accompany them over to another area of the department to try to optimize a sense of privacy before they began communicating with that individual:

The chairs are great, but because there is no place for patients to be moving out of the stretcher, you have people in the chairs, and there is no privacy. You can’t talk and ask people. Sometimes, they are there, I’ll take people around the corner, and I’m talking to them in the hall, just so their neighbour doesn’t hear them, which I personally don’t think it’s appropriate. If they were alone in the chair, I have no problem talking to them, but otherwise, there is no privacy. There is nowhere to sit.

N110

Hopefully, you’re not assessed in the chair. Unless you’re the only person in the chairs at the time, then we would talk to you there, just for privacy reasons. But if there are other people there, we’ve got to take you out of that chair to some corner where we can talk to you privately and then bring you back to the chair.

P201

Participants listed off various areas, wherein the moment, they had sought out a more private location to interact with their patient including a hallway or corner, trauma bay, the resuscitation room, or even another front zone:

Well, they put patients in the chairs when all the [stretcher] beds are full. So, you’re going to see them in the chairs, but there are other people there. I’m not willing to have those conversations unless it’s maybe an infected finger. Even that I really don’t like having in case, there’s something else about it. So, it can be hard to find the space that you can actually talk to somebody. I try to move them around. But you end up going into the quiet room or the resuscitation room or pull them off to the side, trying to see if somebody else’s chairs are empty.

P203

Nurses also acknowledged that although it was an accepted practice to treat patients while they were sitting in a front chair, they were quite uncomfortable doing so when other members of the public were present. During these moments, some nurses admitted that they, too, felt like they were on public display:

I do find there’s far less confidentiality [compared to our old model]. I have to now go into the small area where patients are more or less knee-to-knee with each other, and I have to disclose information or results or do vitals in front of everybody else, spike meds in front of everybody else. You’re being watched, and the patient that you’re doing this stuff to is now the centre focus of everybody in that area.

N104

Two of the nurses who were interviewed described incidents where they felt their privacy was disrespected:

I’ve been caught a couple of times where people are photographing you. That is the culture, and it irritates me because it’s [a mobile device] supposed to be off. And how can we enforce that, when everybody else is on them?

N107

I was on the phone with [details regarding the conversation are anonymized], and then I was called into a patient room and another patient said, “I just wanted to let you know that I feel for you [details regarding what the individual said they overheard are anonymized] and I heard your conversation.” And I'm like, “Oh no. Oh my god.” There’s just no privacy. We have no place to have private phone calls.

N109

## Discussion

### Principal Findings

To our knowledge, this is the first qualitative study to explore the impact of a Lean health care intervention on patient privacy and confidentiality. Although the Lean redesign was intended to make the ED work more efficiently, the results of this study illuminated that the physical configuration of the front cell often intensified the clinical work of emergency nurses and physicians because they needed to actively search for spaces that could better afford privacy and confidentiality for patient encounters.

Evidence-based design of health care facilities requires careful consideration, and anticipation, of the complexities that exist within the delivery of patient care [32]. Although published studies have cautioned against the use of open concept areas in ED settings, as these were associated with increased prevalence of breaches of patients’ informational and physical privacy, the hospital embraced an open concept design for the sets of chairs located in each zone of the front cell. Although professionals did perceive value in having these chairs, they also cautioned that the chairs served multiple, and often competing, purposes. They were part of an active treatment area, they afforded an intermediary space for patients awaiting their results or further diagnostic testing, and as a result of public pressure, they had also become part of a secondary waiting room that housed patients along with their family members and visitors. At any time in the ED, members of the public could fill the front chairs for one or more of these purposes. Again, although previous research had demonstrated the superiority of walled patient areas over those separated by curtains [13,15], when the doctors and nurses in our study interacted with ED patients seated in the front chairs, they were doing so in an area that was absent of any curtains or walls.

Unlike Lin and Lin [17], we found that the ED staff was very vigilant of threats to the ongoing informational and physical privacy of their patients **.** Although nurses were more limited in their ability to work around issues brought on by the configuration of front chairs, professionals were aware that during any given shift, they might need to search for a quieter, more confidential location to engage with their patient. Locating this space was not an easy task to perform when the ED was experiencing a high volume of patients, and physicians noted that their searches for private space could involve temporarily encroaching on another patient treatment area, another front zone, or moving the patient out into a hallway or corridor. Although the conditions that optimized privacy and confidentiality were viewed as being essential for all patients, physicians made a point of highlighting their concerns regarding the vulnerability of individuals who sought medical care from the ED for stigmatized conditions including mental health, addictions, and sexually transmitted diseases. An ED can be the primary source of medical care for patients with stigmatized conditions [33], and although in the moment, an attending may feel that moving a patient out from a front chair into a hallway or corridor may be advantageous to the individuals’ privacy and confidentiality, doing so may actually bring some risk into that encounter. A survey by Stoklosa et al found that 89.5% (206/230) of American emergency physicians believed they deviated from their usual way of performing a physical exam, and 77.5% (286/369) felt they altered how they took a history when they assessed a patient in a hallway. When asked about the impact of these disruptions, over one-third of physicians surveyed admitted they had delays or failures in the diagnosis of hallway-assessed patients, including cases involving psychiatric conditions, substance abuse, and domestic/intimate partner violence [34].

Our study did not focus on change management, and we do not know how closely hospital management has been working with its frontline health care professionals to monitor the ongoing impacts of the restructured ED. Although we do not believe that ED wait times were intentionally privileged over patient privacy, our finding that medical professionals felt the need to move their patients around the department to better afford conditions for their patients’ privacy and confidentiality highlights an important, unintended consequence. Given that Lean assumes that an organization will seek continuous improvement through their examination of whether activities are adding value [20,25], it would seem reasonable that the hospital reflects on how the front chairs have been impacting their ED patients and the nurses and physicians who care for them. We do not know if the hospital is achieving its targets for improved ED wait times, but our participants expressed that during a given shift in the ED, it was common for them to go through the following sequence of activity: request that a patient move out from the front chair area and then ask the patient to accompany them in a search for more private space within the ED; once a suitable spot was located, then the professional interacted with the patient as intended, and then they returned the patient to the front chair area. Professionals viewed this workaround as a way to prevent unintended violations of their patients’ privacy, and thus in the moment, it was viewed by them as being a value-added activity. However, through the lens of the Lean intervention, it may not be a value-added activity. The workaround is likely adding several minutes to the clinical time spent by the professional on that case as well as adding on to the patients’ length of stay in the ED. Our study did not involve discussions with patients, and we cannot make statements regarding their experiences nor perceptions of the quality of medical care they received from the ED. Previous research has shown that although time is important to ED patients, so are other subjective experiences beyond waiting. Patients can show tolerance for waiting when other aspects of their experience were perceived as being well met [35-38]. The question of whether patients value shorter ED wait times over privacy and confidentiality in an ED setting warrants future attention.

The issue of health care professionals being recorded while they provide patient care has been raising concerns within the medical community. About 86% of Canadian households own a cell phone [39], and many members of the public bring these devices with them when they seek medical care [40]. In Canada, hospital policies on cell phone use by the public vary, and there do not appear to be any federal guidelines in place [41].

In terms of patients’ perceptions of, and experiences with, making a cell phone recording in a hospital setting, Oyedokun et al surveyed 110 patients who were treated for a laceration potentially requiring suturing at one of the 3 EDs located in the Canadian province of Saskatchewan [42]. To contrast patient perspectives about recording with the opinions of health care providers, 156 ED professionals (19 nurses and 37 physicians) who practiced at one of the 3 sites were also recruited into this study. Over 80% of patients (81.8%, 90/110) indicated that they had brought a cell phone capable of making a video or audio recording with them to the ED, and 30.8% (33/107) had admitted they contemplated making a video on the day they were surveyed.

Statistically significant differences were found between the proportions of patients versus providers who felt that video recording should be allowed in the ED. Although 61.7% (66/107) of patients were in favor of allowing patients to video record while they were in an ED, 49.5% (51/103) of nurses and 42% (15/35) of physicians indicated that they would allow the patient to do so (chi-square test; *P*<.001). When asked, hypothetically, why they would want to make a video while they were having a suturing procedure performed on them, 43% (24/55) of patients indicated that they would want to do so to be able to share that experience with others, and 38% (21/55) said it would be for a memento of their experience. None of the patients surveyed felt that they would want to video their sutures because they were unsatisfied with the care they had received. Fear of legal action, loss of control over the use and distribution of the video, and feeling that it was generally inappropriate for a patient to make a video during their treatment were among the reasons why providers indicated they would decline their patients’ requests to record.

Although we do not know the contexts under which these incidents occurred, 2 of the nurses in our study spontaneously recalled, respectively, that a patient overheard a conversation that they should not have been privy to and also that another nurse sensed they had been filmed by a member of the public. Both of these nurses felt uncomfortable about what had occurred, and with the findings by Oyedokun et al [42], these incidents continue to raise the question of what degree of informational and physical privacy should be afforded to health care providers. Future research is warranted. At one time, the hospital we studied was noted to have a policy that restricted family members and visitors from being in the front cell. In light of the issues that have been voiced in our study about privacy and confidentiality, it may be time for the ED to revisit the number of members of the public that can be safely, and comfortably, accommodated within patient treatment areas.

### Limitations

This study is not without limitations. As with all studies involving qualitative methodologies, our findings are not generalizable beyond our local context. Exploring the transferability and resonance of our results to other ED settings will require additional research. Given that our study involved discussions with nurses and physicians who provided frontline medical care to patients, we cannot make statements regarding the experiences and opinions of patients who received medical care at the ED nor about the family members and visitors who may have accompanied them. Future research on patients’, family members’, and visitors’ perspectives is needed.

### Conclusions and Implications

To our knowledge, this is the first qualitative study to explore the impact of a Lean health care intervention on the ability of emergency medicine physicians and nurses to optimize conditions for patient privacy and confidentiality. The changes made in the ED included the construction of a three-zone front cell that received all of the patients flowed forward from triage. Each front zone housed an open concept area outfitted with a set of chairs. Our research illuminated that although, in theory, physicians and nurses perceived that the chairs were viewed as adding value to the ED environment, in practice, the chairs served the multiple, and often, competing uses by patients, family members, and visitors. In an attempt to work around the limitations they encountered and keep patients flowing from triage, physicians and nurses revealed that they often needed to move a patient out from a front chair and then go to actively search for another location in the ED that better protected the individual’s informational and physical privacy. These searches involved clinical time and likely impacted the length of stay experienced by some ED patients. We advocate that the physical structure and configuration of the front cell should be re-examined under the lens of Lean’s principle of value-added activities. Future exploration of the perspectives of patients, family members, and visitors regarding the relative importance of privacy and confidentiality during ED care is warranted.

## Abbreviations

ED: emergency department
